# Protecting User Privacy and Rights in Academic Data-Sharing Partnerships: Principles From a Pilot Program at Crisis Text Line

**DOI:** 10.2196/11507

**Published:** 2019-01-17

**Authors:** Anthony R Pisani, Nitya Kanuri, Bob Filbin, Carlos Gallo, Madelyn Gould, Lisa Soleymani Lehmann, Robert Levine, John E Marcotte, Brian Pascal, David Rousseau, Shairi Turner, Shirley Yen, Megan L Ranney

**Affiliations:** 1 Department of Psychiatry University of Rochester Medical Center University of Rochester Rochester, NY United States; 2 Department of Pediatrics University of Rochester Medical Center University of Rochester Rochester, NY United States; 3 Yale School of Management Yale University New Haven, CT United States; 4 Yale School of Public Health Yale University New Haven, CT United States; 5 Crisis Text Line New York, NY United States; 6 Department of Psychiatry and Behavioral Sciences Northwestern University Chicago, IL United States; 7 Department of Psychiatry Columbia University New York, NY United States; 8 Department of Epidemiology Columbia University New York, NY United States; 9 National Center for Ethics in Health Care Veterans Health Administration Washington, DC United States; 10 Harvard Medical School Harvard University Boston, MA United States; 11 Department of Internal Medicine Yale University New Haven, CT United States; 12 Center for Bioethics Yale University New Haven, CT United States; 13 Institute for Social Research University of Michigan Ann Arbor, MI United States; 14 School of Nursing University of Michigan Ann Arbor, MI United States; 15 Center for Internet and Society Stanford Law School Palo Alto, CA United States; 16 Henry J Kaiser Family Foundation Menlo Park, CA United States; 17 Department of Psychiatry and Human Behavior Alpert Medical School Brown University Providence, RI United States; 18 Emergency Digital Health Innovation Program Department of Emergency Medicine, Alpert Medical School Brown University Providence, RI United States

**Keywords:** data sharing, privacy, crisis intervention, text messaging, ethics, business, technology, industry, cooperative behavior, information dissemination

## Abstract

Data sharing between technology companies and academic health researchers has multiple health care, scientific, social, and business benefits. Many companies remain wary about such sharing because of unaddressed concerns about ethics, data security, logistics, and public relations. Without guidance on these issues, few companies are willing to take on the potential work and risks involved in noncommercial data sharing, and the scientific and societal potential of their data goes unrealized. In this paper, we describe the 18-month long pilot of a data-sharing program led by Crisis Text Line (CTL), a not-for-profit technology company that provides a free 24/7 text line for people in crisis. The primary goal of the data-sharing pilot was to design, develop, and implement a rigorous framework of principles and protocols for the safe and ethical sharing of user data. CTL used a stakeholder-based policy process to develop a feasible and ethical data-sharing program. The process comprised forming a data ethics committee; identifying policy challenges and solutions; announcing the program and generating interest; and revising the policy and launching the program. Once the pilot was complete, CTL examined how well the program ran and compared it with other potential program models before putting in place the program that was most suitable for its organizational needs. By drawing on CTL’s experiences, we have created a 3-step set of guidelines for other organizations that wish to develop their own data-sharing program with academic researchers. The guidelines explain how to (1) determine the value and suitability of the data and organization for creating a data-sharing program; (2) decide on an appropriate data sharing and collaboration model; and (3) develop protocols and technical solutions for safe and ethical data sharing and the best organizational structure for implementing the program. An internal evaluation determined that the pilot satisfied CTL’s goals of sharing scientific data and protecting client confidentiality. The policy development process also yielded key principles and protocols regarding the ethical challenges involved in data sharing that can be applied by other organizations. Finally, CTL’s internal review of the pilot program developed a number of alternative models for sharing data that will suit a range of organizations with different priorities and capabilities. In implementing and studying this pilot program, CTL aimed both to optimize its own future data-sharing programs and to inform similar decisions made by others. Open data programs are both important and feasible to establish. With careful planning and appropriate resources, data sharing between big data companies and academic researchers can advance their shared mission to benefit society and improve lives.

## Introduction

Technology companies with large datasets have the potential to fuel discovery in the health sciences and gain valuable insights for their own businesses by sharing their data with academic researchers. Many companies are wary about sharing data with researchers because of concerns about ethics, data security, logistics, and public relations. Previous work published in this journal addresses big data gathered in a health care setting [1,2], but, to date, neither the academic literature nor the technology community has provided guidance for technology companies considering academic data sharing. Without such guidance, few technology companies are willing to take on the potential work and risks involved in noncommercial data sharing, and the scientific and societal potential for their data [3,4] consequently goes unrealized.

In this paper, we describe the 18-month long pilot of a data-sharing program led by Crisis Text Line (CTL), a not-for-profit technology company that provides a free, 24/7 text line for people in crisis in the United States. CTL is the nation’s largest provider of crisis interventions via text. From its inception in August 2013 to January 2018, CTL’s volunteer crisis counselors have conducted more than 1.7 million conversations with 845,545 unique individuals seeking help for a variety of crises, including suicidal behavior, bullying, self-harm, family conflict, and depression (live data depicting trends across texters can be found at CTL’s dedicated tracking website) [5]. The transcripts of these conversations, the metadata they generate (eg, timestamps and area codes), and the postconversation surveys by the crisis counselors (eg, issues encountered and referrals provided) contain rich data about crisis situations. Analyses of these big data have the potential to unearth patterns related both to the needs of youth and adults in crisis and to crisis service delivery, with the attendant possibility of having a wider impact across other fields and organizations that are focused on improving mental health across the world. In addition to sharing insights from its data, CTL has a culture of transparency, continuous learning, and sharing what they have learned in the process of innovation. The information shared in this paper accords with this mission.

CTL wished to find the best ways to share the data they collected with researchers in order to contribute to scientific knowledge, inform mental and public health policy, evaluate and improve the effectiveness of their services, and identify segments of the population that most benefit from CTL services. A pilot data-sharing project was initiated with funding from the Robert Wood Johnson Foundation. The primary goal of the pilot was to design, develop, and implement a rigorous framework of principles that would allow for the safe and ethical sharing of user data, both by CTL and other organizations. CTL’s core priority was to develop policies and procedures that would (1) protect the privacy, confidentiality, autonomy, and well-being of its users and (2) protect the reputation, brand, and public trust of the service—issues that are particularly important for CTL, given the profound sensitivity of the crisis situation data. Existing models from other areas of academia provided helpful templates for data-sharing agreements [6] but were insufficient for addressing the full range of needs, risks, and operational challenges of this project. In addition, CTL wanted to ensure that the results of data sharing were actionable and impactful for the larger community.

In this paper, we describe the process of defining core challenges underlying data sharing in technology-academia partnerships; discuss CTL’s trial solutions to these challenges; and offer lessons learned that might inform other technology companies’ data-sharing partnerships.

## The Crisis Text Line Pilot Program

### Overview

CTL used a stakeholder-based policy process to develop a feasible and ethical data-sharing program, as described below. The stakeholder process comprised forming a data ethics committee; identifying core challenges to address in establishing a data-sharing program; developing open data-sharing principles and protocols; announcing the program and putting in place the necessary infrastructure; iterative refinement of the protocols and infrastructure; launching the data-sharing program; and evaluation of the results of the pilot. It cost approximately US $900,000 to fund the open data-sharing program pilot. This funding covered start-up costs; ongoing technical infrastructure costs; 1 full-time open data manager; as well as engineering, data science, and marketing time.

### The Data Ethics Committee

CTL convened a panel of academic and technology sector experts to form a data ethics committee. Literature reviews and personal recommendations from the CTL advisory board were used to identify researchers with expertise in data security, research ethics, mobile health intervention, and psychology who would be appropriate committee members. The final data ethics committee had 15 members from 13 institutions and was chaired by CTL’s chief data scientist (see Multimedia Appendix 1).

Between January 2016 and April 2017, the data ethics committee met for 4 full group meetings and multiple smaller topic-specific subcommittees via conference call and utilized regular email communications to discuss specific aspects of the data-sharing program. In these meetings, they defined the relevant research ethics concerns, explored options for addressing these concerns, and set criteria for application review. The CTL executive leadership reviewed and approved the policy recommendations put forward by the committee.

### Identifying Challenges

The data ethics committee identified 4 core challenges to address in establishing a data-sharing program. These challenges were research ethics, user confidentiality, data security, and threats to the reputation and operations of the service.

#### Research Ethics

Research on technology-enabled services presents particular opportunities and challenges for protecting the welfare of human subjects [7]; a further set of specific challenges apply in the area of data sharing [8]. For the benefit of organizations that may not be familiar with the principles of research ethics, the 3 core ethical principles highlighted by the data ethics committee were respect for persons (showing regard for individuals’ rights to self-determination and privacy), beneficence (doing no harm and maximizing possible benefits), and justice (ensuring equity in access to research and in protection of vulnerable populations) [7,9].

#### User Confidentiality

The data ethics committee also identified the concept of user data confidentiality (protecting disclosure of identities and information when possible) as critical to a big data-sharing project. Data confidentiality is particularly important for sensitive and potentially stigmatizing data [10], such as that collected by CTL. Large datasets introduce the modern potential challenge of *reidentification* or *deanonymization* of deidentified data provided to third parties: it takes remarkably few pieces of information to identify an individual uniquely, and this task becomes easier the larger and more personalized a dataset is [11]. A further danger is that large datasets may be improperly anonymized [12]. Similarly, blending data from multiple sources also risks deanonymizing the dataset, for the elements in 1 set may fill in the gaps in the other. The CTL data ethics committee was particularly concerned about addressing these potential pitfalls before sharing data.

#### Data Security

Data security refers to protective measures taken by an organization to prevent unauthorized access to computers, databases, and other confidential information as well as to prevent inadvertent disclosure. The highly sensitive nature of the CTL information as well as the vulnerable nature of the CTL client population mandates very high levels of security precautions when using data. Independent technology companies are generally not subject to the Health Insurance Portability and Accountability Act of 1996 [10] Privacy and Security Rules or the Health Information Technology for Economic and Clinical Health Act of 2009 [13]; nor do they have to develop software and hardware according to the Federal Information Processing Standards 140-2 regulations [14,15]. Nonetheless, maintaining adequate data security and user confidentiality safeguards is critical for client trust and is an ethical imperative. In addition, in applications for institutional review board approval before using external companies’ data, academic researchers must demonstrate knowledge of, and adherence to, approved data security best practices for the handling of sensitive and confidential information.

Although all studies should have protocols for avoiding deductive or inadvertent disclosure, highly sensitive data, such as that dealt with by CTL, may require an additional layer of safeguards.

#### Business Challenges

The 2 primary concerns for a company considering sharing data are reputation and cost. Many technology companies holding data that would be useful to science and society are public-facing companies that rely for business success on their reputation or brand, trust from clients or customers, and the loyalty that follows from it. Negative perceptions can spread quickly through modern media and have the potential to cause significant damage in a relatively short period to a company’s image and, by consequence, to its value (as illustrated by recent Facebook scandals).

To mitigate such risks, organizations must calculate and plan for the costs of developing the requisite technical infrastructure and administering a responsible data-sharing program. Technology costs include the development of the data-sharing pipeline and the environment that hosts the data as well as ongoing data hosting fees. Administrative costs comprise principally the costs of staff time and focus. A program manager is required to oversee program marketing, application review, data use agreement execution, and ongoing partner support. Data science and engineering time is required to create custom datasets and offer ongoing technical support.

Although challenges and costs exist, there are many potential benefits that make the investment worthwhile. Collaboration with academic researchers opens up diverse areas of expertise that would be impossible to acquire through internal hiring. In addition, academic-industry partnerships can expand upon the company’s original goals to positively impact their population of users. For example, Twitter’s data-sharing application programming interface (API) has been used to map restaurant violations [16], HIV infection spread [17], and county-level heart disease mortality [18]. These uses of the data might not be at the core of the technology company, yet they align with Twitter’s original mission to “give everyone the power to create and share ideas and information instantly, without barriers.”

### Open Data Program Principles and Protocols

The policy development process described above yielded key principles and protocols for addressing the challenges identified by the data ethics committee and CTL leadership. Table 1 provides a summary of these principles and protocols.

### Technical and Operational Infrastructure and Program Announcement

After developing these policies and procedures, an open data collaborations (ODC) manager was hired by CTL to develop the requisite technical and operational infrastructure for this study and to ensure adherence to the established policies and procedures. The manager’s duties included engaging research teams, coordinating the review of applications, negotiating data use agreements with researchers and their respective universities, developing policies and procedures for sharing custom datasets, and onboarding teams into their data access environments. In addition, the ODC manager, in collaboration with the data ethics committee and the CTL board and staff, developed and iteratively refined the scientific submission and review protocol for applications from academic researchers for data access.

**Table 1:** Open data program: challenge, principles, and protocols.

Challenges and principles		CTL^a^ protocols
**Research ethics**		
	Inform users in an unobtrusive way that anonymized data are shared with select research partners	CTL provides texters with a link to an easy-to-understand Terms of Service^b^, including a disclosure of potential future data use, before every crisis conversation
	Establish a review process that includes outside academics and ethics experts	An internal CTL team and external ethics committee review applications, with special attention paid to nonmaleficence and justice, texter confidentiality, data security, and social impact
	Require human subjects review by academic institution before data sharing	CTL requires each team to procure institutional review board approval
	Ensure adequate protection of marginalized groups	CTL reviews research proposals as well as final manuscripts before journal submission for inadvertent stigmatization of marginalized groups (eg, LGBTQ+)
**User confidentiality**		
	Determine which data are released to each team	CTL creates custom datasets for each team, sharing variables on a need-to-know basis for up to 1 year
	Protect against release of potentially identifying information	In addition to scrubbing all data for personally identifiable information such as names, addresses, emails, and social media handles, CTL transforms or coarsens any data found to pose a risk to texter confidentiality (eg, university name)
**Data security**		
	Maintain possession of and oversight over data and use	CTL gives each team a virtual machine (VM) hosted on Amazon Web Services and accessed via a virtual private network. All analyses are conducted and stored on the VM with copy/paste and export functionalities disabled
	Authorize who can access the data	CTL grants access to university faculty only with demonstration of ethics approval, a signed data use agreement, and a clear data management plan
	Require university oversight of, and liability for, researcher behavior when interacting with the data	CTL signs a Data Use Agreement with the lead researcher as well as his or her respective university
	Limit the total number of teams to allocate sufficient resources, support, and oversight	CTL limits the number of teams to ≤6 per quarter
**Business challenges**		
	Prioritize research that can benefit users and the service	CTL reviews applications for *value* to texters and crisis community. Projects cannot target for-profit ventures or have plans to monetize research output
	Assist with accurate and responsible reporting of results	CTL reviews data output requests and manuscripts before journal submission for accidental breaches of texter confidentiality and accurate contextualization of findings

^a^CTL: Crisis Text Line.

^b^Terms of service: “We have created a formal process for sharing information about conversations with researchers at universities and other institutions. We typically share data with trusted researchers when it will result in insights that create a better experience for our texters. We follow a set of best practices for data sharing based on the University of Michigan’s Inter-University Consortium of Social and Political Research, one of the largest open data projects in the U.S., which includes stringent ethical, legal, and security checks. For more details, see our policies for open data collaborations” [19].

CTL staff first reviewed submissions for team competency; proposal feasibility and value; and texter confidentiality, data security, and research ethics. The data ethics committee then reviewed proposals that had been cleared by the CTL staff, specifically looking for red flags related to texter confidentiality, data security, and research ethics, as well as scientific potential. A standardized (iteratively refined) rubric was used for committee scoring of applications.

In accordance with the developed policies, selected research teams received access to a custom dataset hosted on a virtual data enclave on Amazon Web Services (AWS) servers, where all data storage and analyses took place. Finally, to ensure continued compliance with ethical policies, the ODC manager provided ongoing support (eg, onboarding, technical troubleshooting, and data analysis program installation requests) and reviewed data outputs and publications for accidental breaches of texter confidentiality and accurate data contextualization.

### Refinement of Protocols and Infrastructure

Between September and December 2016, with ongoing consultation from the ethics committee, CTL piloted and reformulated protocols and created and tested the technical infrastructure, including the virtual data enclave and the ability to create custom datasets. Development and maintenance of this infrastructure were time-intensive and had significant start-up and maintenance costs. The initial development phase took 6 months, with an additional 3 months of user testing and refining of the system to meet researchers’ needs. Start-up costs included a call for applications, virtual private network (VPN) hosting, AWS server space, a full-time staff member, and ongoing engineering support. Implementing custom datasets for each research team was also time- and labor-intensive, as it required initial labor to create custom sets and ongoing engineering support for a variety of environments rather than just one.

### Pilot Launch

In February 2016, CTL used its website and press releases to issue the first call to researchers for applications to use the data. The press release generated interest from several media outlets, such as FastCompany and BethKanter.org, which ran feature articles. The data ethics committee accepted applications from research teams on a quarterly basis from April 2016 to April 2017. CTL received over 100 applications across 5 quarterly calls for projects. Following both internal and ethics committee reviews, 20 applications were accepted from teams at 18 different universities. Topics included natural language processing (eg, identifying linguistic markers of help seeking among youth who have been abused), correlational studies exploring mental health drivers (eg, correlating weather patterns to service volume and issue prevalence), service use mapping (eg, mapping service use across the state of Montana to visualize unmet needs and inform future resource allocation), and analysis of specific texter populations (eg, use of service by the LGBTQ+ community). Each team received access to a custom dataset housed within a virtual data enclave, as per protocol. The first teams received access to the data in January 2017. The delay between research project review and access to data was approximately 9 months for the first group of approved projects. By January 2017, the timeline for access to data was approximately 3 months. As of the time of this paper’s writing, none of the studies have published their analyses, although most are reported to have manuscripts in development.

### Refining the Program

As the pilot program was nearing completion, CTL conducted an internal evaluation of the program. CTL used the following criteria to evaluate the success of the program: value (to science and the organization); ethical principles and policies; the ability to share data while maintaining user confidentiality; the ability to provide secure access while maintaining control of data; and the ability to support the program with adequate financial, human, and infrastructure resources. CTL was satisfied that the data-sharing program had value, both to science and to the company, as it prompted greater understanding of data and provided opportunities to share important insights with a broader community. CTL and members of the data ethics committee were also satisfied that the program met its ethical ideals in both principle and practice, that the confidentiality of texters was adequately protected, and that it was feasible to share custom datasets with each research team.

The application process and the data-sharing processes gave CTL confidence that teams were competent, collaborative, and likely to bring value to CTL users and the community at large. These processes provided means to mitigate the user confidentiality and data security risks described above by combining traditional data-sharing processes (eg, use of a VPN and sharing of minimum necessary data for a project) with innovative, technology-specific solutions (eg, a custom-built virtual desktop with safeguards to prevent the copying of data to local machines). However, the financial, infrastructure, and human capital requirements for maintaining a safe, stand-alone open data program were identified as challenging, and the cost and effort of supporting the pilot program were higher than anticipated.

Although start-up funding covered the development of a custom system, maintenance proved to be just as expensive as, if not more than, initial development, as is the case with most rapidly evolving technologies. A related issue is that data hosting is expensive, particularly if each team requires a custom server with custom data. Hosting data for 1 team on AWS servers, for example, costs approximately US $500 a month. Given that the pace of technological change will only increase, CTL identified the difficulties and costs involved in the necessary ongoing auditing and iteration of technical infrastructure as an additional future financial burden. Ultimately, the resources needed to develop and effectively run the data center and related technology significantly outstripped expectations.

In addition to cost, the resources required to oversee the program and provide effective support for research teams were underestimated. In addition to setting teams up with custom data, CTL had to provide ongoing support to add project-specific data analysis software to the virtual server, to clarify aberrations in the data, to troubleshoot bugs, and to review and approve research outputs.

Ethical research collaborations also require communication at every step (data access, custom requests, and output review), and the frequency and difficulty of such communications are amplified when working with researchers from outside the organization. CTL found that the use of human resources and the diversion of focus were higher than originally anticipated. Finally, although free data are desirable to researchers, and therefore drove a high number of applications in this study, researchers found it challenging to focus their time on an unfunded study among their other academic priorities and funded studies. As a result, the pace of research was often slow, with many researchers conducting analyses during weekends and personal time.

On the basis of their internal review, CTL determined that the data-sharing pilot showed promise, but discussion arose about alternatives that might better suit their specific organizational framework and available resources. Resource allocation, both in terms of funding and personnel, is challenging if data sharing is neither part of an organization’s core competency nor a core business objective.

#### Alternative Research Models

In response to the perceived strengths and weaknesses of the initial data-sharing pilot, CTL considered different models for future collaboration with researchers, including alternatives for structuring access to data and for organizing the management of data-sharing projects. The approach pursued in the pilot was designed to maximize the openness of the program by accepting applications from all interested researchers and by approving a large number of projects. This had the advantage of bringing the widest possible range of scholarly perspectives to bear on the data and matched well with CTL’s history of working with crowdsourced capabilities and their commitment to open access. However, it also proved to be a costly model, requiring significant start-up funds and the creation of the necessary technical infrastructure as well as the ongoing provision of tools to allow secure remote access to the data by researchers working across a large number of locations. The required degree of institutional focus was also high, with the program needing a considerable commitment of manpower and attention that, at times, strained CTL’s ability to support its primary mission.

Two alternative, less resource-intensive models were considered. The first involved the funding of a smaller number of resident researchers, who would apply for 3 to 6 months residential fellowships, during which time they would have on-site access to data. This approach would eliminate the cost of developing and maintaining a data center, reduce expenditure of manpower and focus through easing communication and collaboration, and minimize data security concerns. However, this approach would also reduce the number of teams able to work with the data and, thus, the quantity of outputs from the program. In addition, it would restrict access on a geographical basis, limiting participation to those researchers who are physically close enough to take part in the program.

The second alternative involved collaborating closely on an ongoing basis with a select group of trusted research partners. This approach has the advantage of heavily streamlining communications and minimizing distractions from other tasks, while also increasing the organization’s voice both in guiding research questions and in the dissemination of research findings. It also provides financial savings in the maintenance of data-sharing facilities. However, working with a group of trusted partners minimizes the open access nature of the research and, thus, reduces the scope and quantity of research projects.

Ultimately, CTL decided to pursue both alternative strategies concurrently, with each strategy offering a different way of satisfying the underlying principles developed in the first phase of the pilot, often through the implementation of the existing protocols. First, CTL decided to work with very close, highly regarded research partners who would effectively become an extension of the CTL team in the long term. Second, they created an in-house fellowship to allow academic researchers to conduct research via short-term on-site residencies at CTL’s primary location with extremely close oversight. These 2 approaches significantly reduced costs, afforded greater data security, and hold out the promise of increasing opportunities to connect research to outcomes of direct value to the organization.

#### Supplementary Management Tools

Finally, CTL considered 2 different ways of reducing costs and resource usage for data sharing, through the adoption of alternative management models. The first involved using a third-party vendor to manage data warehousing when working with external research partners. This approach reduces the financial and personnel costs of creating and maintaining a data center, increases data security by exploiting the core competencies of the third party, and removes a major attention overhead, thus allowing for an increased focus on leveraging and communicating research outputs. However, because of the necessity of handing over control of highly sensitive data, the organization needs to work with a closely vetted partner. It may also be the case that although small- and medium-sized organizations will reduce their costs by outsourcing the data management element of the program, in very large organizations, it may be possible to scale internal data management solutions to a level that makes the internal option more cost-effective. The revised program adopted by CTL ultimately opted for on-site data access for both in-house research fellows and trusted partners, as the trusted partners were located within easy traveling distance of CTL’s primary site. However, third-party management of remote data access would be compatible with either the original program or a trusted partners program working with partner institutions that are geographically distant.

A second possible option for more efficient management of data-sharing projects is the creation of a separate company dedicated to sharing data. This approach reduces the cost and level of distraction for the parent organization and opens up the possibility of financing data-sharing programs through a different funding model, such as charging an administrative fee for data access, which researchers could build into grant proposals. However, the parent company will still need to provide oversight and consultation for the spin-off company because staff in the parent company will have a unique view into the data and their meaning. CTL ultimately decided that their needs could be met through the other revisions made to their program and, thus, opted not to pursue the spin-off company structure.

## Guidelines for Organizations Considering Data Sharing

CTL created a trial data-sharing program both to explore how their collection of data could generate new benefits for their users and to assist other helping organizations through providing access to CTL’s data and feedback as well as to the lessons learned in the innovation process. Although it proved feasible and ethical to create the pilot program, the cost of sustaining this particular model was ultimately too high in the light of other organizational priorities. However, this set of principles provided a secure foundation on which CTL was able to iterate further in the development of new data-sharing programs. The principles also offer a framework that other organizations can now adapt to create safe and ethical programs of their own, allowing the broader sharing of data while protecting the privacy of users.

On the basis of our experiences, we suggest that organizations considering data sharing with academic researchers pursue the approach outlined, which has been reverse engineered from the questions studied by CTL during their evaluation phase.

### Determine the Value and Suitability of the Data and Organization

The first step for organizations considering academic data sharing is to establish (1) whether the data they have collected are, in principle, valuable to science and (2) whether academic study of the data will further the goals of the organization and/or, in the case of profit-oriented companies, whether study of the data will add value to the business. Once it is determined that suitable data are available for sharing, the following 4 questions need to be answered to determine whether an ethically rigorous and practically feasible program can be established:

- Do we have access to research and ethics expertise to review policies, protocols, and proposals?
- Are our data of such a type that they can be deidentified effectively and shared with researchers in a manner that protects user confidentiality?
- Can we offer secure portals for accessing data?
- Do we have the financial resources, the human capital, and the physical and digital infrastructure to support ethical sharing of data without undermining other organizational priorities?

**Figure 1:**
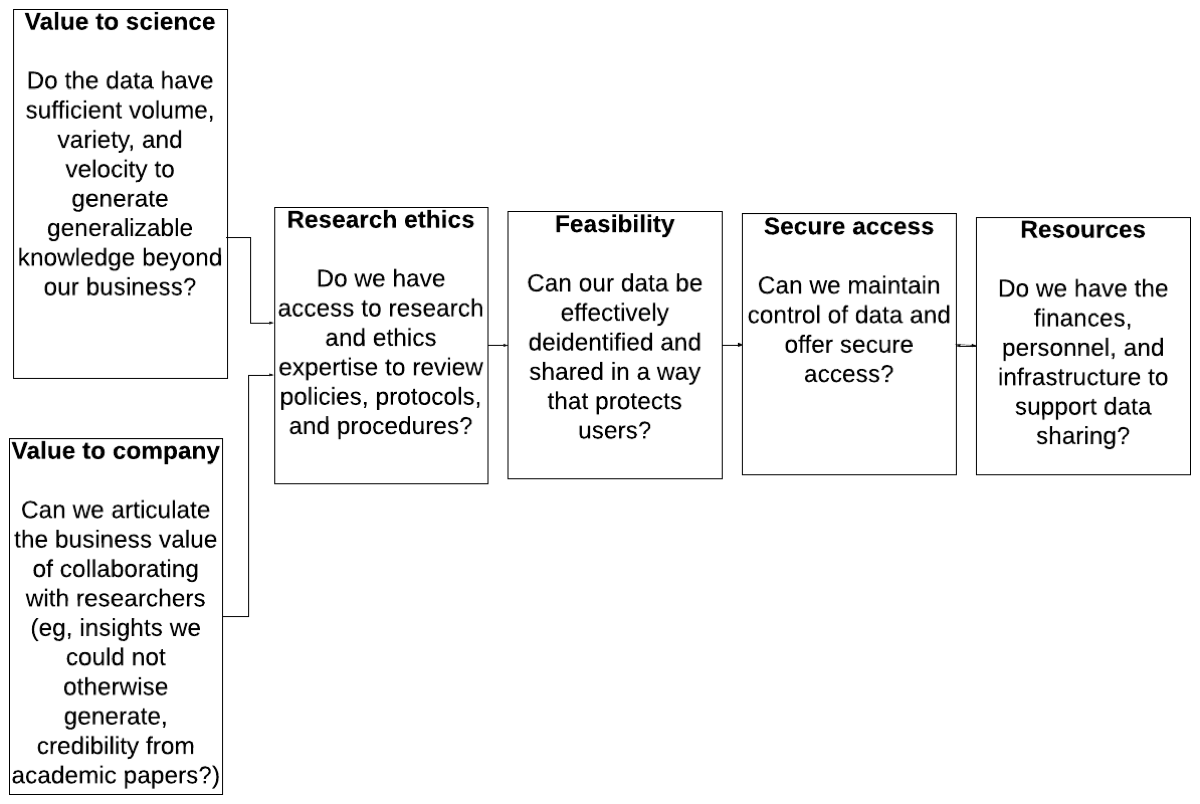
Key questions for organizations considering data sharing.

If all of these questions yield affirmative answers, the organization can then move on with confidence to developing the policies that will guide their particular data-sharing program (see Figure 1 for a summary).

### Decide on an Appropriate Data Sharing and Collaboration Model

In developing its data-sharing program, CTL engaged in a rigorous process of review that examined the pros and cons of the initial pilot program and then studied 2 further potential program structures. The 3 different models considered in this study, along with their advantages and disadvantages, are provided in Table 2. Organizations considering data sharing can draw on CTL’s experiences to help identify the model that is most appropriate for them.

**Table 2:** Research partnership models.

	Open data-sharing program (pilot)	Resident researchers	Selective research partnership
Summary	Open application process for multiple teams to access data and conduct diverse studies at a distance	Researchers apply for 3 to 6 months on-site residency with access to data via computers maintained by the organization	Collaborate closely with a select few trusted research partners on a long-term ongoing basis
Pros	Maximizes variety and quantity of research projects	Eliminates cost of developing data center; eases communication and collaboration; and reduces data security concerns	Increases the organization’s voice in guiding research questions and operating principles and increases control over dissemination of research findings
Cons	Most costly option, requiring both start-up and maintenance funding and personnel	Geographic limitation to research collaborators and fewer teams at once	More limited scope of research

### Develop Protocols and Technical Solutions for Safe and Ethical Data Sharing and the Best Organizational Structure for Implementing the Program

Once an appropriate data-sharing model has been selected, it will be necessary to draw on the available research and ethics expertise to develop policies and protocols that match the principles of the organization, and then to review research proposals. The pilot conducted by CTL has provided a transferable set of principles (see Table 1, column 1) and a prototype for protocols (see Table 1, column 2). Each organization will have to determine how to apply these principles in their own particular niche (see Table 3).

**Table 3:** Data management models.

	Internal data management	Third-party data management
Summary	The organization manages data warehousing and access solutions	A third-party vendor manages the data warehousing for an external partner
Pros	Provides maximum control over data security and increases responsiveness to needs of the organization and researchers	Reduces technical costs of starting and maintaining a data center; increases data security, given the third party’s core competencies; and enables focus on leveraging and communicating research outputs
Cons	Significant expenses involved in starting and maintaining a data center and draws focus away from organization’s core competencies	Organization loses some control over data, therefore must work with a vetted partner

It is not a small thing to institute a program of this sort. Organizations that decide to create a data-sharing program will need to decide how much energy and resources they can reasonably devote to it and assess how the implementation of the project will affect their overall mission. If creating the data solution, or even the overall oversight structure, will become too overwhelming in terms of resources or focus, organizations might consider outsourcing data management or even spinning off a separate organization (for-profit or nonprofit) to reduce these burdens.

### Conclusions

The implementation and evaluation of the CTL open data collaboration pilot provides a planning model for *big data* technology companies interested in collaborating with academics. This extends previous work on human subject concerns [7] and on big data in health care [1,2] by focusing specifically on the ethical and practical considerations for data sharing and collaboration in prevention research. We identified key principles in the areas of research ethics, user confidentiality, data security, and business challenges and then developed innovative protocols to ensure that these principles governed the manner in which data were shared. The lessons learned in this process, and in the evaluation of the pilot model, have been distilled into a set of guidelines that can be used by other organizations considering their own academic data-sharing programs.

Big data are generated all the time from a myriad of sources, each with its own potential value to science and its own ethical challenges. We believe that the principles and protocols offered here can be applied and adapted to a broad variety of contexts. However, some limitations should be noted. First, the data collected by CTL are, for the most part, of such a type that users are aware of what they are sharing. In other contexts, such as data collection from passive sources, eg, Global Positioning System sensors, researchers must pay additional attention to the right to privacy of users as well as to obtaining informed consent. Second, although the volume of data generated by CTL is extremely high, some technology companies have datasets that are even larger and more complex. Organizations wishing to share these larger datasets will face additional challenges. Potential research teams will need to be vetted to ensure that they have the technical and data science capabilities to make the best possible use of these datasets. In parallel, the organization will also need to ensure that data are presented in a format that will be as easy as possible for researchers to handle.

On the basis of both the successes and challenges of the CTL data-sharing pilot, we urge organizations that are considering sharing data with academic researchers to evaluate their preparedness carefully and determine which models are most suitable for their specific institutional needs. In addition to the type of internally managed open data-sharing program trialed by CTL, technology companies may also wish to consider other research partnership models, such as those summarized in Table 2, and other approaches to data management, such as those in Table 3. Key variables to consider include the company’s objectives for data sharing, the degree of control they wish to maintain, and their available financial and staffing resources.

Open data programs are important and feasible to establish. Companies embarking on such projects should be aware of the significant commitments and responsibilities involved in the sharing of data. With careful planning and appropriate resources, data sharing between big data companies and academic researchers can advance their shared mission to benefit society and improve lives.

## Appendix

Multimedia Appendix 1 Crisis Text Line data ethics committee members.

## Abbreviations

API: application programming interface
AWS: Amazon Web Services
CTL: Crisis Text Line
ODC: open data collaborations
VPN: virtual private network
