# Correction: Protecting User Privacy and Rights in Academic Data-Sharing Partnerships: Principles From a Pilot Program at Crisis Text Line

**DOI:** 10.2196/67880

**Published:** 2024-12-20

**Authors:** JMIR Editorial Office

**Affiliations:** 1 JMIR Publications Toronto, ON Canada

**Keywords:** conflicts of interest, disclosure

In “Protecting User Privacy and Rights in Academic Data-Sharing Partnerships: Principles From a Pilot Program at Crisis Text Line” (J Med Internet Res 2019;21(1):e11507) [1], a change has been made to the Conflicts of Interest section.

According to a commentary by Tim Reierson [2], the following relationships were originally not explicitly declared in the Conflicts of Interest section (author initials were added by the editor for clarity):

One other author was a Crisis Text Line staff member, the study’s open data collaborations manager Nitya Kanuri. Her staff member affiliation is undeclared in the paper. The following affiliations to Crisis Text Line, Inc. were also undeclared: the corresponding author [AP] and one other author [MG] were serving on the clinical advisory board to Crisis Text Line. Seven other authors [BP, DR, JEM, LSL, MLR, RL, SY] were members of the Data Ethics Committee to the study. The Data Ethics Committee was listed on Crisis Text Line’s official website as an advisory panel of experts to the organization. [2]

The corresponding author Pisani, in their response [3], argues that “far from being ‘undisclosed,’ the participation and collaboration of members of the author group with Crisis Text Line in documenting the results of their pilot academic data sharing program was a key feature of the article” and further adds that “an appendix was provided listing members of the data ethics committee, which included several of the authors.”

We agree with this characterization. We do not see any attempts to deceive reviewers or editors by concealing that this is a collaborative project that involved staff members of Crisis Text Line and academic experts and ethicists comprising the Data Ethics Committee, who advised Crisis Text Line on best practices on data sharing with researchers, which was the topic of the viewpoint article.

However, JMIR Publications’ policy has stated since 2017 that conflicts of interest disclosures for authors include “non-financial competing interests,” including “membership in a government or other advisory board” [4]. This is consistent with the guidance from the International Committee of Medical Journal Editors (ICMJE) on conflicts of interest disclosures [5].

Given the Loris.ai controversy, which unfolded after publication of the 2019 paper, and which involved sharing of anonymized Crisis Text Line data with the commercial subsidiary Loris.ai for machine learning purposes [6], these relationships as well as relationships to Loris.ai could have been disclosed more explicitly, which is why we are updating the Conflicts of Interest section for this publication accordingly.

Crisis Text Line emphasizes that Loris.ai did not exist during the time of the academic data sharing pilot [7]. From a publication ethics perspective, the most relevant question is whether any of the authors (including the three coauthors affiliated with Crisis Text Line) benefitted or had the potential to benefit financially from Loris.ai, at the time of submission or acceptance of their article. As Reierson points out:

None of the authors made any disclosures or acknowledged the existence of the for-profit or its licensing agreement with Crisis Text Line. Loris.ai, Inc. had been in operation for months by the time the paper was submitted for publication. [2]

In response [3], the authors assured us that “no one on the CTL [Crisis Text Line] data advisory board had a role in or even knew about Loris.ai.” In addition, we received confirmation from the three authors who were staff members of Crisis Text Line (Shairi Turner, Current Chief Health Officer at Crisis Text Line; Bob Filbin, former Chief Data Science officer at Crisis Text Line who left in 2021; and Nitya Kanuri, former Open Data Collaborations Manager at Crisis Text Line) that they did *not* have a financial stake in Loris.ai. (NK did not include Crisis Text Line as an affiliation because that author was affiliated with Crisis Text Line only in 2016-2017, and they had moved on by the time when the paper was published; past affiliations are often not listed, and there is a lack of a consensus in the scholarly publishing industry on what affiliation should actually be declared upon submission [8,9]).

Any financial stake in Loris.ai certainly should have been disclosed with the 2019 paper published in the *Journal of Medical Internet Research*, but would not necessarily invalidate the viewpoint article (which focuses on a *noncommercial* sharing scenario). While Crisis Text Line staff Bob Filbin and Shairi Turner conceded that they had knowledge of the license agreement between Loris.ai and Crisis Text Line at the time of submission, it is an open question whether such knowledge could have biased the work of the independent Data Ethics Committee. However, we are satisfied to hear that the Data Ethics Committee, which evidently spearheaded the guidelines and processes for noncommercial academic data sharing, did not know about Loris.ai and did not benefit from any commercial data sharing agreement personally, nor did any of the authors personally benefit from such an arrangement financially.

JMIR Publications’ policy on conflicts of interest follows ICMJE recommendations [4,5] and includes procedures relating to post-publication handling of undisclosed conflicts of interest [10]. As a member of the Committee on Publication Ethics (COPE), the *Journal of Medical Internet Research* adheres to COPE guidance on these matters and hereby updates the Conflicts of Interest section of the published viewpoint article by Pisani et al [1], as follows:

**Conflicts of Interest**

(updated December 2024) NK was a salaried full-time employee with Crisis Text Line in 2016-2017, overseeing the Robert Wood Johnson Foundation–funded, open data pilot described in this article; they were no longer in that role at the time of publication of this article. BF was a salaried Chief Data Scientist at Crisis Text Line and chaired the Data Ethics Committee. ST served as Chief Medical Officer for Crisis Text Line and received a salary. AP and MG were uncompensated members of the clinical advisory board to Crisis Text Line. As outlined in Multimedia Appendix 1, several of the authors (BP, DR, JEM, LSL, MLR, RL, and SY) served as uncompensated members of the Data Ethics Committee for Crisis Text Line. None of the authors reported any financial benefits from a data sharing agreement between Crisis Text Line and its for-profit subsidy Loris.ai, including a financial stake in Loris.ai. None of the authors except Crisis Text Line leaders (BF and ST) had contemporaneous knowledge of a license agreement between Crisis Text Line and Loris.ai. The data sharing agreement between Crisis Text Line and Loris.ai was terminated in 2022.

The *Journal of Medical Internet Research* editors do not think that this revised conflicts of interest statement affected the peer review process or the validity of this viewpoint article. Consequently, the noted issue of concern regarding undisclosed conflicts of interest, given the information currently available, did not appear to meet criteria for further action, such as a notice of editorial concern or retraction [11].

The correction will appear in the online version of the paper [1] on the JMIR Publications website on December 20, 2024, together with the publication of this correction notice. Because this correction was made after submission to PubMed, PubMed Central, and other full-text repositories, the corrected article has also been resubmitted to those repositories.
