# Supplementary material for: Protecting User Privacy and Rights in Academic Data-Sharing Partnerships: Principles From a Pilot Program at Crisis Text Line
Source: J Med Internet Res. 2019 Jan 17;21(1):e11507. doi: 10.2196/11507 (PMC6354196; doi:10.2196/11507)
Supplement: Multimedia Appendix 1 [file jmir_v21i1e11507_app1.pdf]

Crisis Text Line Data Ethics Committee Members

Brian Pascal, JD.
Fellow, Center for Internet and Society, Stanford Law School
David Rousseau, MPH.
Vice President and Executive Director of Health Policy Media and Technology, The Henry J. Kaiser Family Foundation
Dominic Sisti, PhD.
Assistant Professor of Medical Ethics and Health Policy; Assistant Professor of Psychiatry; Director, The Scattergood Program for Applied Ethics of Behavioral Health Care, the Hospital of the University of Pennsylvania
Jeremy N. Block, PhD, MPP.
Assistant Professor of Population Health Science and Policy; IRB Chair, Icahn School of Medicine at Mount Sinai; Scholar, Healthcare Innovation Technology Laboratory (HITLAB)
John E. Marcotte, PhD.
Director, Data Sharing for Demographic Research & Data Security Officer, Inter-university Consortium for Political and Social Research (ICPSR), University of Michigan
John Wilbanks
Chief Commons Officer, Sage Bionetworks; Senior Fellow, FasterCures
Lisa Lehmann, MD, PhD, MSc.
Executive Director, National Center for Ethics in Health Care, U.S. Department of Veterans Affairs; Associate Professor of Medicine and Medical Ethics, Harvard Medical School; Associate Professor of Health Policy and Management, Harvard T.H.Chan School of Public Health
Lucy Bernholz, PhD.
Senior Research Scholar, Digital Civil Society Lab, Stanford University; Visiting Scholar, Center on Philanthropy and Civil Society, Stanford University
Megan L. Ranney, MD, MPH.
Assistant Professor of Emergency Medicine, Assistant Professor of Health Services, Policy and Practice, Brown University; Director, Brown Emergency Digital Health Innovation program
Rebecca Weintraub Brendel, MD, JD.
Clinical Director at the Red Sox Foundation and Massachusetts General Hospital Home Base Program; Assistant Professor of Psychiatry, Harvard Medical School
Robert J. Levine, MD.
Professor of Medicine, Yale School of Medicine; Senior Research Scholar and Chair: Executive Committee, Interdisciplinary Center for Bioethics, Yale University
Sandra Soo-Jin Lee, PhD.
Senior Research Scholar, Pediatrics, Center for Biomedical Ethics, Stanford School of Medicine; Faculty, Program in Science, Technology and Society, Stanford University
Shalon M. Irving, PhD, MPH, CHES.
Epidemic Intelligence Service (EIS) Officer, Centers for Disease Control and Prevention
Shirley Yen, PhD.
Associate Professor (Research), Department of Psychiatry and Human Behavior, Alpert Brown Medical School
Stephanie Alessi Kraft, JD.
Senior Fellow, Treuman Katz Center for Pediatric Bioethics, Seattle Children’s Research Institute
